# A letter from a patient: Awareness regarding medical errors and patient engagement

**DOI:** 10.1002/jgf2.598

**Published:** 2022-11-28

**Authors:** Takashi Watari, Krista Votruba

**Affiliations:** ^1^ General Medicine Center Shimane University Hospital Shimane Japan; ^2^ Department of Medicine University of Michigan Medical School Ann Arbor Michigan USA; ^3^ Patient Overland Park Kansas USA

On October 11, 2022, I received a thoughtful email from Miss Krista Votruba from Kansas, US. The event she described to me changed my thinking regarding patient engagement. Although nearly all medical errors involve patients themselves, many patient safety interventions have focused on the traditional models of changing provider behavior, promoting interprofessional collaboration, and reinforcing a culture of safety.^1^ In Japan, papers on patient engagement in patient safety are limited, and even in many clinical society meetings, we have had little opportunity to explore the experiences, perspectives, and opinions of patients themselves.^2^, ^3^ We have even perhaps overlooked the role of patients in safety activities in medicine. Here is her true patient experience. Unexpected adverse events may occur in isolated computed tomography and magnetic resonance imaging (MRI) examination chambers. I draw your attention, as patient safety experts, to a paper on MRI skin burns from 4 years prior.^4^ Krista read the paper carefully and contacted me and stated that it completely matched her own patient experience. On September 26, 2022, she experienced invisible painful skin burns after MRI. She was wearing functional exercise clothing but was not instructed to change before the scan. The radiologic technician told her she could wear her shoes, socks, and clothes during the MRI. Further, the technician did not turn on the microphone and did not give her the emergency button. Since the technician did not place her appropriately in the examination chamber, her skin was touching the skin at her underarms. Her right shoulder was placed in a holder to image her right rotator cuff. Furthermore, the technician inserted earplugs in her ears and put headphones with music on her. Shortly after the scan started, during the first song, her body felt warm. During the second song, she felt extremely hot; however, she realized that she did not have the emergency button. Therefore, she started yelling and kicking her feet to draw the technician's attention, but it went unnoticed. She managed to maneuver her right shoulder out of the holder and slide it as far to the left as she could, but this also went unnoticed. When the fourth song was playing, the technician finally came and let her out of the machine.

When she removed her sweatshirt, she felt that her body was on fire. Therefore, she went to the dressing room and checked her back for burn marks, but there were none. For the next 24 h, she experienced severe burning and felt like her insides were on fire. She went to the University of Kansas Burn Unit, where the nurse practitioner told her that there is no such thing as an MRI burn. After 24 h of feeling as if her insides were on fire, the burning started to move to her skin. She still felt a burning sensation from her feet to her head, but this was unobservable to anyone else. The only visible indication was the bright red skin on her chest and neck, which looked sunburned. She went to urgent care and was prescribed a steroid cream to be mixed with aloe for her skin for 3 days. She subsequently went to her primary care doctor, who ordered a laboratory workup, which showed an elevated inflammation level. She has been taking ibuprofen and using aloe to cool her skin. The burning pain on her skin surface continued over 4 weeks. However, because no other visual signs of general burns (as medical personnel might expect) were present, she stated that she was not taken seriously by the staff. This is her true experience as a patient, and we, as medical professionals, can learn a lot from the patient's story. These details correspond to the typical illness of MRI thermal burn injury previously reported.^4^ Additionally, from both these experiences, the risk of burns may not be well known in the US, and medical staff are not knowledgeable regarding wearing functional exercise clothes during MRI examinations. Krista informed me that she had researched various scientific papers herself. However, the risk is well known among radiologic technicians working at academic centers in Japan, and some precautionary measures are normally taken. However, owing to the lack of information in English, this information is possibly not widely disseminated. Recently, functional garments have become popular in the market.^5^ However, asking patients to change their clothes before MRI to prevent skin burns may not be common practice among professionals in some countries.

This is just the tip of the iceberg, and this case only focuses on one area. There are probably countless underlying causes of medical errors in everyday practice (but not so much every day for patients) that we medical professionals are not aware of. The best way to understand them and reduce the number of patients experiencing similar causes is for primary healthcare providers to take errors seriously based on patient‐centered principles and take the occurrence of adverse events as a learning experience. Most importantly, patients should be involved in detecting adverse events, empowering them to ensure the safe delivery of care. We believe that this cannot be accomplished without emphasizing patient involvement as a way of improving the culture of safety.

## AUTHOR CONTRIBUTIONS

All authors had access to the information used, and all authors participated in the preparation of this manuscript.

## FUNDING INFORMATION

None declared for all authors.

## CONFLICT OF INTEREST

None declared for all authors.

## PATIENT CONSENT STATEMENT

The patient provided consent for publication of this letter.

## Data Availability

All the relevant data are contained in the report.
